# Balancing of sulfur storage in maize seed

**DOI:** 10.1186/1471-2229-12-77

**Published:** 2012-05-30

**Authors:** Yongrui Wu, Wenqin Wang, Joachim Messing

**Affiliations:** 1 Waksman Institute of Microbiology, Rutgers University, 190 Frelinghuysen Road, Piscataway, NJ, 08854, USA

**Keywords:** Sulfur, Methionine, Cysteine, RNAi, Nutrition and maize

## Abstract

**Background:**

A balanced composition of amino acids in seed flour is critical because of the demand on essential amino acids for nutrition. However, seed proteins in cereals like maize, the crop with the highest yield, are low in lysine, tryptophan, and methionine. Although supplementation with legumes like soybean can compensate lysine deficiency, both crops are also relatively low in methionine. Therefore, understanding the mechanism of methionine accumulation in the seed could be a basis for breeding cultivars with superior nutritional quality.

**Results:**

In maize (*Zea mays*), the 22- and 19-kDa α-zeins are the most prominent storage proteins, nearly devoid of lysine and methionine. Although silencing synthesis of these proteins through RNA interference (RNAi) raises lysine levels in the seed, it fails to do so for methionine. Computational analysis of annotated gene models suggests that about 57% of all proteins exhibit a lysine content of more than 4%, whereas the percentage of proteins with methionine above 4% is only around 8%. To compensate for this low representation, maize seeds produce specialized storage proteins, the 15-kDa β-, 18-kDa and 10-kDa δ-zeins, rich in methionine. However, they are expressed at variant levels in different inbred lines. A654, an inbred with null δ-zein alleles, methionine levels are significantly lower than when the two intact δ-zein alleles are introgressed. Further silencing of β-zein results in dramatic reduction in methionine levels, indicating that β- and δ-zeins are the main sink of methionine in maize seed. Overexpression of the 10-kDa δ-zein can increase the methionine level, but protein analysis by SDS-PAGE shows that the increased methionine levels occur at least in part at the expense of cysteines present in β- and γ-zeins. The reverse is true when β- and γ-zein expression is silenced through RNAi, then 10-kDa δ-zein accumulates to higher levels.

**Conclusions:**

Because methionine receives the sulfur moiety from cysteine, it appears that when seed protein synthesis of cysteine-rich proteins is blocked, the synthesis of methionine-rich seed proteins is induced, probably at the translational level. The same is true, when methionine-rich proteins are overexpressed, synthesis of cysteine-rich proteins is reduced, probably also at the translational level. Although we only hypothesize a translational control of protein synthesis at this time, there are well known paradigms of how amino acid concentration can play a role in differential gene expression. The latter we think is largely controlled by the flux of reduced sulfur during plant growth.

## Background

The main storage proteins in maize, also called zeins, are encoded by a multigene family and divided into four classes, i.e., α (19- and 22-kDa), γ (50-, 27- and 16-kDa), β (15-kDa) and δ (18- and 10-kDa) zeins. The general feature of all zein proteins in amino acid composition is that they are nearly lysine-free and very rich in glutamine and proline. However, each class is characteristic for their specific amino acid biases. The γ-zeins are abundant in cysteine, whereas δ-zeins contain very high percentages of methionine [1,2]; β-zein is rich in both [3], whereas α-zeins are devoid of them.

Breeders, maize geneticists, and molecular biologists have applied different strategies for generating maize inbreds with a balanced amino acid composition. High-lysine maize mutants with an opaque phenotype were identified based on the reduction of lysine-free α-zein proteins and a compensatory increase of non-zein proteins [4]. For instance, *opaque 2* (*o2*) lines of maize could contain nearly twice the amount of lysine compared to normal maize depending on the genetic background [5]. Still, *o2* maize could not be commercialized, because of its soft kernel texture and high sensitivities to insects and diseases. These adverse properties have been overcome with the introduction of quantitative trait loci (QTLs) that restore kernel hardness even with reduced levels of α-zein proteins, which is recognized by the reversion of the opaque to normal seed phenotype. Because of these improvements, CYMMIT, who developed these maize lines, coined them Quality Protein Maize (QPM) [6]. Interestingly, the introgressed QTLs raise the expression of γ-zeins, which appear to be able to restore kernel hardness despite the reduced levels of α-zeins [7]. Today, QPM has been introduced into 23 developing countries and grown over 10 million acres. Using dominant RNA interference (RNAi) to reduce α-zeins instead of using the recessive *o2* mutation [8-10], can be used for advanced breeding of QPM and simplify its broader geographical application [10].

In developed countries, like the United States, QPM is not widely grown, because maize is not the main source of protein. As feed, maize is always supplemented with soybean, which contains sufficient levels of lysine. However, like maize, soybean is also deficient in methionine so that the animal diet is further fortified with chemically synthesized methionine, which is a racemic mixture of L- and D-methionine. This not only adds billions of dollars in cost every year, but the health impact of a racemic mixture has also been raised. Whereas the lack of lysine in α-zeins can be compensated with increased levels of non-zein proteins in the seed, this shift cannot take place because of the low representation of sulfur amino acids in proteins in general. Moreover, *o2* mutants have a reduced β-zein level [11], which in turn results in even lower methionine levels [5,12]. Because of these properties, a different strategy will be needed for increasing both lysine and methionine in maize to levels that avoid costly supplementation. However, unlike high-lysine mutants that have a visible phenotype, variation of methionine levels among inbred lines does not produce a visible phenotype. On the other hand, a biochemical seed germination screen, selecting for resistance to feedback inhibition of the biosynthesis of methionine, has been used to identify maize inbred BSSS53 having elevated levels of methionine in its seeds [12] that was due to the enhanced expression of the 10-kDa δ-zein gene [13,14]. This link between seed methionine levels and expression of high-methionine storage proteins was further illustrated with the ectopic expression of 10-kDa δ- and 15-kDa β-zein genes in different species to study their stability and spatial deposition in heterologous system [15,16]. When the 10-kDa δ-zein gene was overexpressed in maize, the methionine level was significantly increased, close to that of BSSS53 [17]. Other approaches that increase the synthesis or reduce the metabolism of methionine have also been exploited in other species [18-20].

Our hypothesis is that seed storage proteins serve as a sink for photosynthates. During their growth plants convert reduced elements of nitrogen and sulfur into amino acids using energy from photosynthesis. This scheme is easy to follow for reduced nitrogen based on the rebalancing that occurs when zeins are reduced and non-zeins are elevated [21]. However, the mechanism for the storage of sulfur-amino acids (cysteine or methionine) is less clear. Because seed proteins have evolved specialized proteins for cysteine and methionine accumulation and storage, we reasoned that knock-downs of different zein proteins through RNAi could shine new light on the reduced sulfur sink in the seed. Indeed, when we reduced protein rich in cysteine, protein with methionine is increased, consistent with their biochemical pathway.

## Results

### Comparison of amino acid composition in different zeins

**Figure 1:**
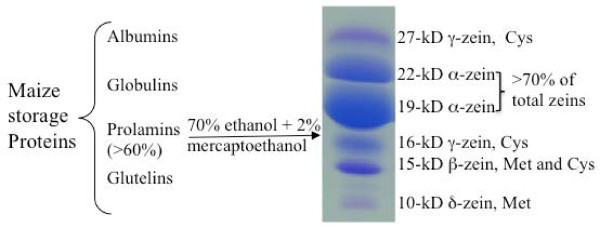
**Classification of maize storage proteins.** Protein isolation and gel electrophoresis are described under Methods.

Storage proteins are grouped into albumins, globulins, glutelins, and prolamins (the latter also called zeins in maize) based on their solubility in different solvents (Figure 1) [22]. Total maize storage proteins are made of more than 60% zeins, of which about 70% are α-zeins [23]. The α-zeins are lysine-free and also low in methionine with less than 1% (Table 1). The others are also deficient in lysine with the exception of the 50-kDa γ-zein. However, the 50-kDa γ-zein is expressed at very low levels and only contains 2.52% lysine (Table 1). Therefore, it does not contribute to total lysine levels in the seed in any significant way. In general, γ-zeins are abundant in cysteine and δ-zeins in methionine, whereas β-zein is rich in both cysteine and methionine (Table 1). In B73 seed flour, the lysine, methionine and cysteine levels are 2.54%, 2.30% and 2.40%, respectively (Table 1), all significantly lower than an average of 5%, a level at which the 20-L amino acids would be balanced in composition from a nutritional point of view.

**Table 1:** Biased amino acid composition in zein protein sequences of different classes

**Zeins**	**MW (kDa)**	**Accession No.**	**Mature Peptide**	**Met%**	**Cys%**	**(Met + Cys%)**	**Lys%**
α	22 (*fl2*)	L34340.1	241	0.00%	0.41%	0.41%	0.00%
	19 A	BT034568.1	219	0.94%	0.94%	1.88%	0.00%
	19 B	DQ244961.1	220	0.46%	0.91%	1.37%	0.00%
	19 D	BT061340.1	219	0.46%	0.46%	0.92%	0.00%
γ	50	AF371263	278	1.08%	5.40%	6.48%	2.52%
	27	AF371261	204	0.49%	7.35%	7.84%	0.00%
	16	AF371262	163	1.84%	7.36%	9.20%	0.00%
β	15	AF371264	160	11.25%	4.38%	15.63%	0.00%
δ	18	AF371265	190	25.26%	1.58%	26.84%	0.53%
	10	AF371266	129	22.48%	3.88%	26.36%	0.00%
Total Protein^a^	/	/	/	2.30%	2.40%	4.70%	2.54%

^**a**^Data was measured from B73 seed flour. It was calculated by the percentage of an amino acid in total seed protein.

### The main sink of methionine in maize seed

The contribution of the methionine-rich β- and δ-zeins to entire methionine levels in seed was evaluated in inbred A654 background (see Methods), because it carries natural null alleles of both 18- and 10-kDa δ-zein genes [14]. The absolute amino acid level (AA_ab_) is calculated by the amount of an amino acid in total seed flour, whereas the relative level (AA_rel_) is represented by the percentage of an amino acid in total protein. The latter determines the nutritional value of seed protein.

**Figure 2:**
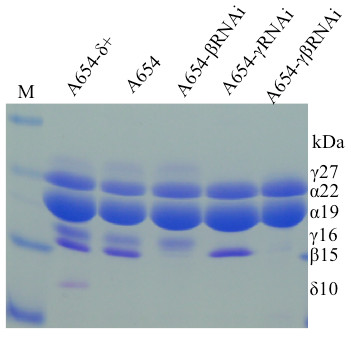
**Zein accumulation pattern in inbred A654 and its derivative lines with intact δ-zein alleles, βRNAi, γRNAi or both RNAis.** Protein from 500 μg of maize flour was loaded in each lane. M, protein markers from top to bottom being 37, 25, 20, 15 and 10 kDa. The size of each zein band is indicated with numbers in the “kDa” column.

Both Met_ab_ and Met_rel_ in A654 were about 20% lower than those in A654-δ (Figure 2 and Table 2). After introduction of an RNAi event against β-zein in A654 (Figure 2), Met_ab_ and Met_rel_ dropped even further to 38% and 40%, respectively (Table 2), indicating that β- and δ-zeins provided for methionine sinks in the seed. The specificity of this drop could be shown, when an RNAi event directed against γ-zeins was layered on top of the β-zein-RNAi. No further reduction in the Met_ab_ and Met_rel_ levels was noticed because γ-zeins are low in methionine. On the other hand, Cys_ab_ and Cys_rel_ were dramatically decreased by 30% and 26%, respectively (Table 2), indicating that γ- and β-zeins provided for cysteine sinks in the seed. As expected, Lys_ab_ and Lys_rel_ were elevated in A654-βγRNAi, since lysine-free zeins were suppressed, whereas proteins with normal lysine levels were elevated (Table 2).

**Table 2:** Amino acid composition in A654 and its different RNAi mutants

	**A654-δ**		**A654**		**A654-βRNAi**		**A654-γβRNAi**	
**Amino acids**	**AA**_**ab**_	**AA**_**rel**_	**AA**_**ab**_	**AA**_**rel**_	**AA**_**ab**_	**AA**_**rel**_	**AA**_**ab**_	**AA**_**rel**_
Methionine	0.24%	2.42%	0.20%	2.09%	0.15%	1.44%	0.13%	1.40%
Cysteine	0.23%	2.32%	0.24%	2.51%	0.22%	2.12%	0.16%	1.72%
Lysine	0.23%	2.32%	0.23%	2.41%	0.25%	2.40%	0.29%	3.12%
Total protein	9.90%		9.55%		10.40%		9.30%	

### The difference between nitrogen and methionine sinks

**Figure 3:**
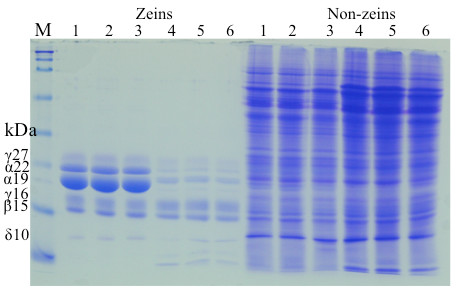
**Protein accumulation pattern of seeds from the cross of B73 x*P6z1CRNAi***. Lanes 1–3, vitreous kernels with genotype *B73/-; −/−*; lanes 4–6, opaque kernels with genotype *B73/-; P6z1RNAi/-*. Zein and non-zein accumulation patterns of *B73/-; −/−* and B73/-; P6z1RNAi/- were analyzed by 15% SDS-PAGE. Protein from 500 μg of maize flour was loaded in each lane. M, protein markers from top to bottom being 250, 150, 100, 75, 50, 37, 25, 20, 15 and 10 kDa. The size of each zein band is indicated with numbers in the “kDa” column.

The mechanism underlying the elevated lysine level in mutants with reduced accumulation of zeins is based on the fact that the reduction of lysine-free zeins is compensated by an increase of lysine-balanced non-zein proteins, resulting in nearly unchanged total protein level but enhanced lysine content (Figure 3). Because α-zeins, which amount to about 70% of total zeins, are also deficient in methionine (Table 1), one would expect the same compensation mechanism for methionine. However, when 19-and 22-kDa α-zeins are knocked down with a double RNAi construct, neither Met_ab_ nor Met_rel_ increase significantly, whereas Lys_ab_ and Lys_rel_ were elevated by 59% and 56%, respectively (Table 3). Therefore, either non-zeins are lower in methionine than lysine or a different mechanism controls the accumulation of methionine.

**Table 3:** Amino acid composition in z1RNAi seed flour

				
	**B73/-; −/−**		**B73/-; P6z1RNAi/-**	
**Amino acids**	**AA**_**ab**_	**AA**_**rel**_	**AA**_**ab**_	**AA**_**rel**_
Methionine	0.22%	2.22%	0.24%	2.38%
Cysteine	0.24%	2.42%	0.24%	2.38%
Lysine	0.27%	2.73%	0.43%	4.26%
Total protein	9.90%		10.10%	

Currently, 36,201 protein-coding sequences with amino acids longer than 100 residues have been annotated in the maize database (http://www.plantgdb.org/search/misc/plantlistconstruction.php?mySpecies=Zea%20may s). By calculating the amino acid composition in each protein sequence, it was found that about 57% of the proteins have lysine residues above 4%, whereas only about 8% of the proteins have methionine residues above 4%. Proteins with both lysine and methionine above 4% only take up about 5% (Table 4). The relative lower content of methionine than lysine in predicted protein sequences then makes it plausible that raising the non-zein protein level cannot balance the methionine sink in the seed.

**Table 4:** Amino acid composition in maize protein sequences

**Total protein sequences**	**36, 201**	**100%**
Lys (>4%)	20, 509	57%
Met (>4%)	3, 017	8%
Cys (>4%)	2, 977	8%
(Lys Met) (>4%)	1, 825	5%

### Linkage of high-methionine phenotype to suppression of cysteine-rich zeins

**Figure 4:**
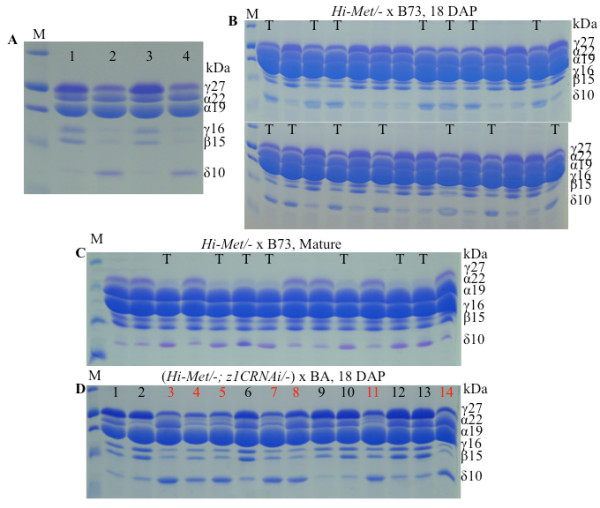
**Zein accumulation patterns of Hi-Met transgenic seeds.A,** Hi-Met transgenic seeds in lane 2 and 4 accumulate significantly higher amounts of the 10-kDa δ-zein, but their β- and γ-zeins are lower than non-transgenic controls in lanes 1 and 4. **B,** linkage analysis of the Hi-Met transgene and the suppression of β- and γ-zeins in immature seeds from Hi-Met/- x B73 at 18 DAP. The kernels inheriting the Hi-Met transgene expressing higher level of the 10-kDa δ-zein are marked with “T.” **C,** as described in B, but seeds are at maturity. **D,** linkage analysis of the Hi-Met transgene and the suppression of β- and γ-zeins in the progeny from the cross of (Met/-; z1CRNAi/-) x (Hi-II B x A). The kernels inheriting the Hi-Met transgene expressing higher level of the 10-kDa δ-zein are marked red. M, protein markers from top to bottom being 37, 25, 20, 15 and 10 kDa. The size of each zein band is indicated with numbers in the “kDa” column.

Consistent with these results is the overexpression of the methionine-rich 10-kDa δ-zein, the resultant transgene called Hi-Met, in which the methionine-rich 10-kDa δ-zein was overexpressed under the control of the strong 27-kDa γ-zein promoter [17]. Western blot analysis, indeed, confirmed enhanced 10-kDa δ-zein and total methionine levels [17]. We then could ask, whether elevated levels of the δ-zein would induce any compensatory adjustment among other zeins. Therefore, total zeins were extracted from the Hi-Met line and subjected to SDS-PAGE. Interestingly, the expression of the three cysteine-rich 27-kDa, 16-kDa γ- and 15-kDa β-zeins was noticeably reduced in Hi-Met seeds compared to non-transgenic controls, whereas the accumulation of the cysteine-poor α-zeins was unchanged (Figure 4A). To examine whether the high 10-kDa δ-zein level phenotype was genetically linked to the low expression of cysteine-rich zeins, a heterozygous transgenic plant (*Hi-Met/-*) was pollinated with B73 pollen. Single mature and developing seeds from 18-DAP were then analyzed for zein accumulation phenotypes (Figure 4B and C). Indeed, the high and low expression phenotypes of 10-kDa δ-zein segregated with a ratio of 1:1. Furthermore, the high expression phenotype of the 10-kDa δ-zein was linked to the suppression of γ- and β-zeins (Figure 4B and C).

As a control, we used the transgenic maize z1CRNAi event that reduced 22-kDa α-zeins [8]. When a plant with the genotype *Hi-Met/-; z1CRNAi/-* was pollinated with non-transgenic pollen of a High-II B x A cross, all progeny inheriting the transgene *Hi-Met* accumulated reduced γ- and β-zeins (Figure 4D). Therefore, the linkage between high δ-zein and low β- and γ-zeins was independent of the accumulation of α-zeins.

### Linkage of knock-downs of cysteine-rich proteins to high-methionine phenotype

**Figure 5:**
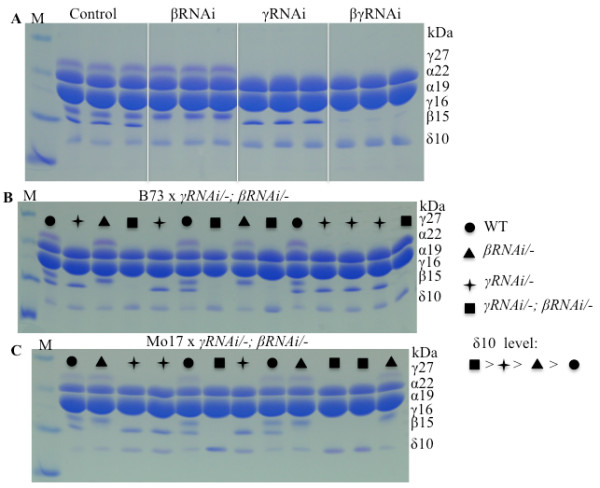
**Increased accumulation of the 10-kDa δ-zein in transgenic RNAi seeds.A,** Comparison of the 10-kDa δ-zein level in non-transgenic control, βRNAi, γRNAi and βγRNAi in Hi-II background. **B,** linkage analysis of the 10-kDa δ-zein level and RNAis in seeds from the cross of B73 x γRNAi/-; βRNAi/-. **C,** linkage analysis of the 10-kDa δ- zein level and RNAis in seeds from the cross of Mo17 x γRNAi/-; βRNAi/-. Kernel genotypes are marked with different symbols. M, protein markers from top to bottom being 37, 25, 20, 15 and 10 kDa. The size of each zein band is indicated with numbers in the “kDa” column.

Cysteine and methionine are the only two amino acids containing sulfur among the 20 L-amino acids. The enzymes cystathionine γ-synthase (CGS) and cystathionine β-synthase constitute the committing steps in the transfer of the sulfur moiety from cysteine to methionine. If the flux of sulfur to methionine were interrupted because of the increased use of cysteine in protein synthesis, would such a block slow the flux of sulfur to methionine and in turn reduce protein synthesis that consumes methionine? Indeed, using RNAi constructs that reduce the expression of cysteine-rich zeins [24], βRNAi, γRNAi, and βγRNAi increase proportionally the accumulation of the 10-kDa δ-zein (Figure 5A). In progeny from the cross of B73 or Mo17 x β*RNAi*/-; γ*RNAi*/- kernels with both RNAi genes accumulated the highest level of the 10-kDa δ-zein. Progeny inheriting γ*RNAi* or β*RNAi* had an inverse proportional reduction of their mRNAs, correlating directly to their cysteine codons, respectively, whereas the progeny inheriting neither of the RNAi genes had the lowest 10-kDa δ-zein levels (Figure 5B and C). Therefore, it appears that the level of β- and γ- zeins can regulate the downstream methionine sink.

## Discussion

### Layers of regulation of the methionine level in maize seeds

**Figure 6:**
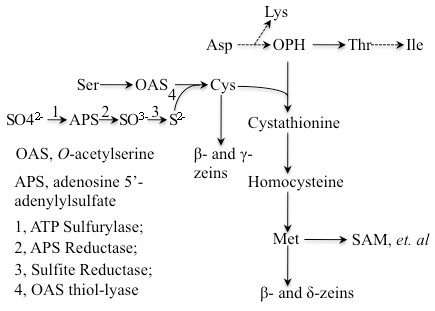
**Pathways for sulfate reduction and synthesis of cysteine and methionine.** The essential amino acids lysine, threonine, isoleucine and methionine are synthesized from the same precursor aspartic acid. Methionine is a sulfur-containing amino acid, in which the sulfur moiety is transferred from cysteine by CGS. The sulfide in cysteine is reduced from sulfate absorbed from the soil by three steps as shown in the diagram.

Sulfur is one of the essential elements in organisms. During plant growth sulfur and nitrogen are taken up from the soil, reduced, and incorporated into amino acids during photosynthesis (Figure 6). The intermediate product is cysteine, from which methionine, the only other sulfur-containing amino acid, and a variety of other compounds receive reduced sulfur, such as SAM, glutathione and sulfolipids [19,25]. During senescence amino acids are transported to the seeds to capture the recovered energy from reduced nitrogen and sulfur for the next generation. Because seeds have low levels of free amino acids during desiccation, they have to store them in proteins compatible with seed dormancy and germination. It appears that reduced nitrogen can be sunk into proteins in general as protein synthesis can balance the reduction of storage proteins with non-storage proteins. However, cysteine and methionine have to be captured in a different way because there are only two amino acids out of twenty with sulfur. Furthermore, these two amino acids, occur in rather low frequencies in proteins because of their specialized function, as one can see from the comparison of the presence of lysine, methionine, and cysteine in the coding regions of the maize genome (Table 4). Interestingly, maize embryos contain an average lysine but very low methionine level, whereas the endosperm has higher methionine but lower lysine levels [12]. Lysine levels have been substantially increased in the presence of RNAi directed against α-zeins in endosperm [8-10,21], but methionine levels were only slightly elevated under these conditions (Table 3). In *o2*, a mutant of the transcription factor that controls a subset of α-zeins, the methionine level was even lower than that in normal inbreds [5,12], probably due to the decreased accumulation of β-zein.

Therefore, it is intriguing that methionine is concentrated in just a few storage proteins that are synthesized during endosperm development. However, maize seed methionine level varies among different inbred lines ranging from deficiency to a level that does not require fortification with the addition of synthetic methionine in animal feed [26]. Among the tested lines, BSSS53 contains the highest level of methionine. This trait in BSSS53 segregates with the expression levels of the 10-kDa δ-zein, providing us with a genetic link between the 10-kDa δ-zein gene and the methionine sink in the seed. Furthermore, different alleles of δ-zein genes and alleles of regulators of δ-zein genes that act posttranscriptionally rather than transcriptionally correlate with this trait directly [2,14,27].

### Sulfate reduction limits further increase in methionine level

Heterologous systems have already been used to investigate zeins as sulfur sink. For instance, the 15-kDa β-zein gene has been overexpressed in tobacco and a legume species alfalfa to evaluate its ability to store reduced sulfur if amino acid biosynthesis is raised [28,29], with the assumption that free methionine might be limiting in the synthesis of β-zein in alfalfa. Indeed, it was found that co-expression of the cystathionine γ-synthase from *Arabidopsis thaliana* (*AtCGS*) and the β-zein gene raised the levels of β-zein transcript and protein, confirming that β-zein was posttranscriptionally regulated by free methionine levels [29].

These observations in alfalfa are consistent with the results shown here that β-and δ-zeins represent the major sink of methionine, and β- and γ-zeins of cysteine in maize seed (Table 2). Overexpression of the 10-kDa δ-zein increases the methionine level, but decreases β- and γ-zeins at the same time, which could trigger a threshold limit for raising methionine levels. In fact, although Hi-Met transgenic maize contained significantly higher methionine levels than the non-transgenic control, it did not exceed natural levels already found in inbred BSSS53 [17]. Moreover, if further increases of methionine levels would occur at the expense of cysteine due to β- and γ-zeins (Figure 5), low levels of those would pose other seed deficiencies. Indeed, it has been shown that γ-zeins are essential for endosperm modification to maintain kernel vitreousness in quality protein maize (QPM) [7].

Because methionine is derived from cysteine, serving as the thiol moiety donor, it suggests that the bottleneck for the flux of sulfur is sulfur uptake and reduction to increase amino acid biosynthesis during plant growth. The combined action of three enzymes takes sulfur from the oxidation state +6 to −2, catalyzed by ATP sulfurylase, APS reductase (APR) and sulfite reductase, respectively (Figure 6). The reduced sulfide then reacts with *O*-acetylserine (OAS), forming the end product of assimilation of cysteine catalyzed by OAS thiol-lyase. OAS is formed by serine acetyltransferase (SAT). Cysteine not used in the synthesis of cysteine-rich proteins, like β- and γ-zeins, could flow into methionine and induce the translation and thereby the stability of the 10-kDa δ-zein mRNA (Figure 5). Such a flow could potentially be achieved by overexpressing the committing enzyme APR or SAT during photosynthesis. Indeed, heterologous expression of bacterial APR has been shown to increase expression of the δ-zein protein expression [30]. Alternative strategies for increasing cysteine biosynthesis could also be achieved with the overexpression of SAT [25].

## Conclusion

In contrast to the nitrogen sink, storage of sulfur is controlled through the accumulation of a few specialized proteins in maize endosperm. We propose that β- and δ-zeins are the major sink for methionine and β- and γ-zeins for cysteine in the seed of maize. Because methionine constitutes the endpoint of the sulfur amino acid biosynthetic pathway and the sulfur moiety goes through cysteine, increased expression of methionine-rich proteins starves the accumulation of cysteine-rich proteins during endosperm development. Therefore, we hypothesize that the major bottleneck for increased seed methionine may be the reduction of sulfur in the leaves during photosynthesis.

## Methods

### Genetic stocks

The βRNAi, γRNAi, z1CRNAi, P6z1RNAi and Hi-Met transgenic plants in this work were all generated in our lab and have been described before [8,10,14,17,24]. Inbred line A654 is a natural null mutant for the 18-kDa and 10-kDa δ-zein genes. A654-δ was generated by introgressing two intact δ-zein alleles from B73, whereas A654-βRNAi, A654-γRNAi, and A654-βγRNAi were produced by crossing the corresponding RNAi lines with A654 first. All resulting materials were backcrossed to A654 for two generations and then selfcrossed for two or three generations to generate homozygous intact δ-zein alleles. βRNAi in A654-βRNAi should also be homozygous because it has no phenotype. A654-γRNAi was semi-opaque and A654-βγRNAi completely opaque, so that A654 inheriting γRNAi or both βRNAi and γRNAi was easy to score.

### Total zein and non-zein protein extraction, protein and amino acid composition analysis

For zein extraction, the dry kernels were wrapped individually in two layers of thick aluminum foil and crushed into fine flour with a heavy hammer and immature seeds at 18 DAP were frozen in liquid nitrogen and ground in a mortar. For segregation analysis, kernels were ground individually. Only 50 mg (mature seed) or 100 mg (18 DAP) of flour was transferred to a 2 ml Eppendorf tube, then mixed and vortexed with 400 μl of 70% ethanol/2% 2-mercaptoethanol (v/v), then kept on the bench at room temperature overnight; the mixture was centrifuged at 13,000 rpm in a benchtop microfuge for 10 min, then 100 (mature seed) or 200 (18 DAP) μl of the supernatant liquid was transferred to a new tube; 10 μl of 10% SDS was added to the extract, the mixture was dried by vacuum and resuspended in 100 μl of distilled water.

For extraction of non-zein from dry seeds, the supernatant from above was discarded. Solids remaining in the tube were resuspended with zein extraction buffer to completely remove the zeins from other proteins. This step was repeated for three times. At last, the residual solids were suspended in 400 μl of non-zein extraction buffer (12.5 mM sodium borate, 5% SDS and 2% 2-mercaptoethanol (vol/vol)). The mixture was kept at 37°C for two hours and vortexed several times during this period. The mixture was centrifuged at 13,000 rpm for 10 min, and then 100 μl of the non-zein supernatant was transferred to a new tube. 4 μl (equal to 500 μg of flour) of each sample was analyzed with 15% SDS-PAGE gel, run at 200 Volts for 35 min. The resulting gel was stained with Commassie buffer.

About 20 g of mature seeds were ground to fine flour. The protein and amino acid composition analysis was conducted by the New Jersey Feed Laboratory, Inc., Trenton, NJ, USA.

### Genome-wide analysis of amino acid composition in maize protein sequences

The *Zea mays* database comprises a comprehensive, high quality, and freely accessible resource of 42,654 protein sequences that were downloaded from PlantGDB http://www.plantgdb.org/search/misc/plantlistconstruction.php?mySpecies=Zea%20mays. A self-written Perl-script was made to calculate Lys, Met, and Cys ratio in each protein sequence longer than 100 amino acids. Among 36,201 protein sequences with more than 100 amino acids, only the proteins with more than 4% Lys, Met, or Cys were identified and counted.

## Authors’ contributions

YW, WW and JM designed the experiments and analyzed the data. YW and JM interpreted the data and wrote the manuscript. All authors read and approved the final manuscript.
